# Isoamylcyanoacrylate for sutureless horizontal rectus recession surgery

**DOI:** 10.4103/0301-4738.77030

**Published:** 2011

**Authors:** V P Gupta, Pragati Gupta, Rigved Gupta

**Affiliations:** Department of Ophthalmology, University College of Medical Sciences and GTB Hospital, Delhi - 110095, India; 1 Department of Ophthalmology, LHMC, New Delhi, India; 2 Department of Ophthalmology, University College of Medical Sciences and GTB Hospital, Delhi - 110095, India

Dear Editor,

We read the article by Darakshan *et al*.[1] with keen interest. We wish to make following observations to refine this article:

1. The use of tissue adhesives as an alternative to sutures in strabismus surgery is not new.[2] Most of the studies are in experimental animals.[2–4] Only few studies of cyanoacrylate in strabismus surgery in humans are on record.[1,5,6] We congratulate the authors for reporting cyanoacrylate in rectus muscle recession for strabismus in India. However, authors[1] missed to cite the article which was the first one to describe the use of cyanoacrylate in strabismus surgeries in 10 patients.[6]
2. The authors have mentioned that one drop of isoamylcyanoacrylate (IAC) was applied to both the scleral site and the cut edge of the muscle and held them in apposition.[1] While performing this, two problems can arise. First, if the cut edge of the tendon is not adequately adhered to the sclera and the muscle sheath gets strong adhesion with the sclera, this situation may result in the retraction of muscle fibers within the sheath clinically manifesting as consecutive strabismus. Second, the possibility of the spread of the adhesive beneath the arc of contact by capillarity exists considering that such substances are liquid and lead to the complete adherence of the whole arc of contact.[4,5] In such an unexpected event, the postoperative result may mimic the recession with Faden operation. Mulet *et al*. solved this problem by including additives in the adhesive mixture to give it higher viscosity.[5] Authors have not addressed these concerns.
3. Authors did not mention the cases in which the attachment failed and IAC was reapplied or switched to sutural recession surgery.
4. The number of exotropes and esotropes in Table 1 are 6 and 4, which is not similar to that mentioned in results.
5. Authors failed to mention the cases in which the safety suture was not removed. It would be impossible to rule out whether the safety suture acted as hang-back recession in the case of an unrecognized slipped muscle.
  In contrast to this study, the duration of surgery using cyanoacrylate was faster than suture in experimental studies.[3] While estimating the price, the cost of 8-0 vicryl used for conjunctival closure was not considered. One vicryl suture may be adequate for two muscle recessions in experienced hands.
6. The authors did not comment upon the efficacy of the procedure with respect to mean pre- and postoperative deviation and postoperative correction: percentage of orthophoria, over- or undercorrection, ocular motility, muscle shift, and slipped muscle. Mulet *et al*. reported undercorrection in 30% eyes (3/10) of the bioadhesive group requiring second surgery.[5] Cyanoacrylate adhesives may produce an imprecise final position for the reinserted rabbit muscle, because of the growth of muscle fibers anterior to the point of gluing.[4]
  Lastly, none of the patients had any complication in this study. The observer bias in favor of IAC cannot be ruled out in their study. The postoperative follow-up should have been performed by an independent observer unaware of the IAC / suture groups. Cyanoacrylates may cause various complications such as erosion, ulceration, and areas of necrosis in surrounding tissues, giant papillary conjunctivitis, granulomatous keratitis, cataracts, and retinal toxicity.[5] This trial did not evaluate conjunctival scarring/ulcer, scleral ulceration / perforation, anterior/posterior scleral whitening, granuloma, and retinal whitening. Granulomas were observed in 20% of muscle recessions using the adhesive.[5] Postoperative slit-lamp biomicroscopy and indirect ophthalmoscopy should have been done to detect above complications.
